# Case report: Duplication of the *GCK* gene is a novel cause of nesidioblastosis: evidence from a case with Silver-Russell syndrome-like phenotype related to chromosome 7

**DOI:** 10.3389/fendo.2024.1431547

**Published:** 2024-12-10

**Authors:** Takashi Shoji, Ichiro Yamauchi, Hidenori Kawasaki, Kogoro Iwanaga, Takuro Hakata, Daisuke Tanaka, Junji Fujikura, Toshihiko Masui, Hisato Suzuki, Mamiko Yamada, Kenjiro Kosaki, Yosuke Kasai, Etsuro Hatano, Akira Inaba, Takahito Wada, Shinji Kosugi, Yohei Ueda, Toshihito Fujii, Daisuke Taura, Nobuya Inagaki

**Affiliations:** ^1^ Department of Diabetes, Endocrinology and Nutrition, Kyoto University Graduate School of Medicine, Kyoto, Japan; ^2^ Department of Genomic Medicine, Kyoto University School of Public Health, Kyoto, Japan; ^3^ Department of Pediatrics, Kyoto University Graduate School of Medicine, Kyoto, Japan; ^4^ Department of Surgery, Kurashiki Central Hospital, Kurashiki, Japan; ^5^ Center for Medical Genetics, Keio University School of Medicine, Tokyo, Japan; ^6^ Department of Surgery, Kyoto University Graduate School of Medicine, Kyoto, Japan; ^7^ Clinical Genetics Unit, Kyoto University Hospital, Kyoto, Japan; ^8^ Medical Research Institute KITANO HOSPITAL, PIIF Tazuke-kofukai, Osaka, Japan

**Keywords:** nesidioblastosis, Silver-Russell Syndrome, hyperinsulinemic hypoglycemia, chromosome 7, glucokinase

## Abstract

Silver-Russell syndrome (SRS) is a syndrome characterized by prenatal and postnatal growth retardation, facial features, and body asymmetry. SRS is often complicated with hypoglycemia, whose etiology is unclear. We describe the clinical course of 25-year-old man with hypoglycemia. We diagnosed him with hyperinsulinemic hypoglycemia (HH) and treated him with laparoscopic distal pancreatectomy. Histological examination led to a diagnosis of nesidioblastosis. The juvenile onset of his nesidioblastosis and its slowly progressive course suggested a genetic etiology. Whole-exome sequencing (WES) identified the heterozygous *NR0B2* Ala195Ser variant, which alone was unlikely to cause nesidioblastosis because this variant is sometimes detected in the Japanese population. Copy number analysis using WES data suggested duplication in chromosome 7, and subsequent G-banding chromosome analysis confirmed mos dup(7)(p11.2p14). We determined that the patient had SRS-like phenotype based on his clinical features and this duplication. Furthermore, we found that the duplicated region contained the *GCK* gene, whose gain-of function variants could cause HH. Taken together, the patient’s HH may have been caused by duplication of the *GCK* gene, which could be a novel cause of nesidioblastosis.

## Introduction

Silver-Russell syndrome (SRS) is an imprinting disorder that causes growth retardation, characteristic facial features, and body asymmetry (1). The Netchine-Harbison clinical scoring system (NH-CSS) is used to diagnose SRS based on six features: small for gestational age, postnatal growth failure, relative macrocephaly at birth, protruding forehead, body asymmetry, and feeding difficulties and/or low body mass index (2). While the clinical features of SRS are well known, the genetic background is complicated. Loss of methylation of chromosome 11p15 (11p15 LOM) is observed in 30–60% of patients with SRS (1), while maternal uniparental disomy of chromosome 7 (upd(7)mat) is seen in 10%. Patients with SRS due to upd(7)mat often present with less typical features than those due to 11p15 LOM (3, 4). In addition to its usual characteristics, SRS is often accompanied by hypoglycemia. Previous reports demonstrated that 15–26% of patients with SRS experienced hypoglycemia (4, 5). The pathogenesis of hypoglycemia in this context is unclear, but it is thought to involve feeding difficulties and growth hormone insufficiency (6).

Hypoglycemia has a variety of causes in adults. Drugs (e.g., insulin and insulin secretagogue), clinical illnesses, hormone deficiencies (e.g., glucocorticoids, growth hormone, and thyroid hormone), and non-islet cell tumors should be considered. Endogenous hyperinsulinemic hypoglycemia (HH) is also an important differential diagnosis (7). A key pathophysiological feature of HH is inappropriate secretion of insulin when fasting plasma glucose levels are low (7). The diagnosis is based on the results of the classical 72-hour fasting test: plasma insulin level ≥ 6 µU/mL, plasma C-peptide level ≥ 0.2 nmol/L, and plasma proinsulin level ≥ 5 pmol/L at end-of-fast glucose < 45 mg/dL (8). The majority of adult-onset HH is caused by insulinoma, whose estimated prevalence is 0.16 per 100,000 population (9).

Other causes of HH in adults include functional β-cell disorders, gastric surgery, and insulin autoimmune hypoglycemia. Functional β-cell disorders belong to a group of ill-defined entities (10). Among them, nesidioblastosis is histologically characterized by β cells with enlarged hyperchromatic nuclei and abundant clear cytoplasm (11). Congenital hyperinsulinism (CHI) is a common cause of HH during infancy and childhood. Several genes causing CHI have been identified: *ABCC8, KCNJ11, GCK, GLUD-1, HADH1, SLC16A1, HNF1A, HNF4A UCP2, HK1, PGM1*, and *PMM2* (12).

Here, we report an adult male patient with hypoglycemia who was diagnosed clinically and pathologically with HH due to nesidioblastosis. Genetic analyses showed that he had somatic mosaicism for segmental duplication of 7p11.2–p14. We reviewed his clinical history and phenotype, and then determined that he had SRS-like phenotype. Although previously reported patients with SRS-like phenotype caused by duplication of similar sites also had hypoglycemia (13) and hyperinsulinemia (14), the causes were unclear. The patient in the present case was confirmed to have HH, which suggests that duplication of the *GCK* gene located in 7p11.2–p14 is a novel cause of HH.

## Case description

### Clinical course

A 20-year-old male patient was incidentally found to have asymptomatic hypoglycemia. His plasma glucose level was 59 mg/dL and his immunoreactive insulin (IRI) level was 20.6 µU/mL. His plasma glucose level after a 72-hour fast was 64 mg/dL. He was referred to Kyoto University Hospital for further examination. As no pancreatic tumors were detected by contrast-enhanced computed tomography (CT), magnetic resonance imaging (MRI), or endoscopic ultrasonography (EUS), we began careful follow-up. During the follow-up period of 5 years, his plasma glucose levels gradually decreased and his body weight increased by 5 kg. We decided to re-evaluate his hypoglycemia at 25 years of age.

**Figure 1:**
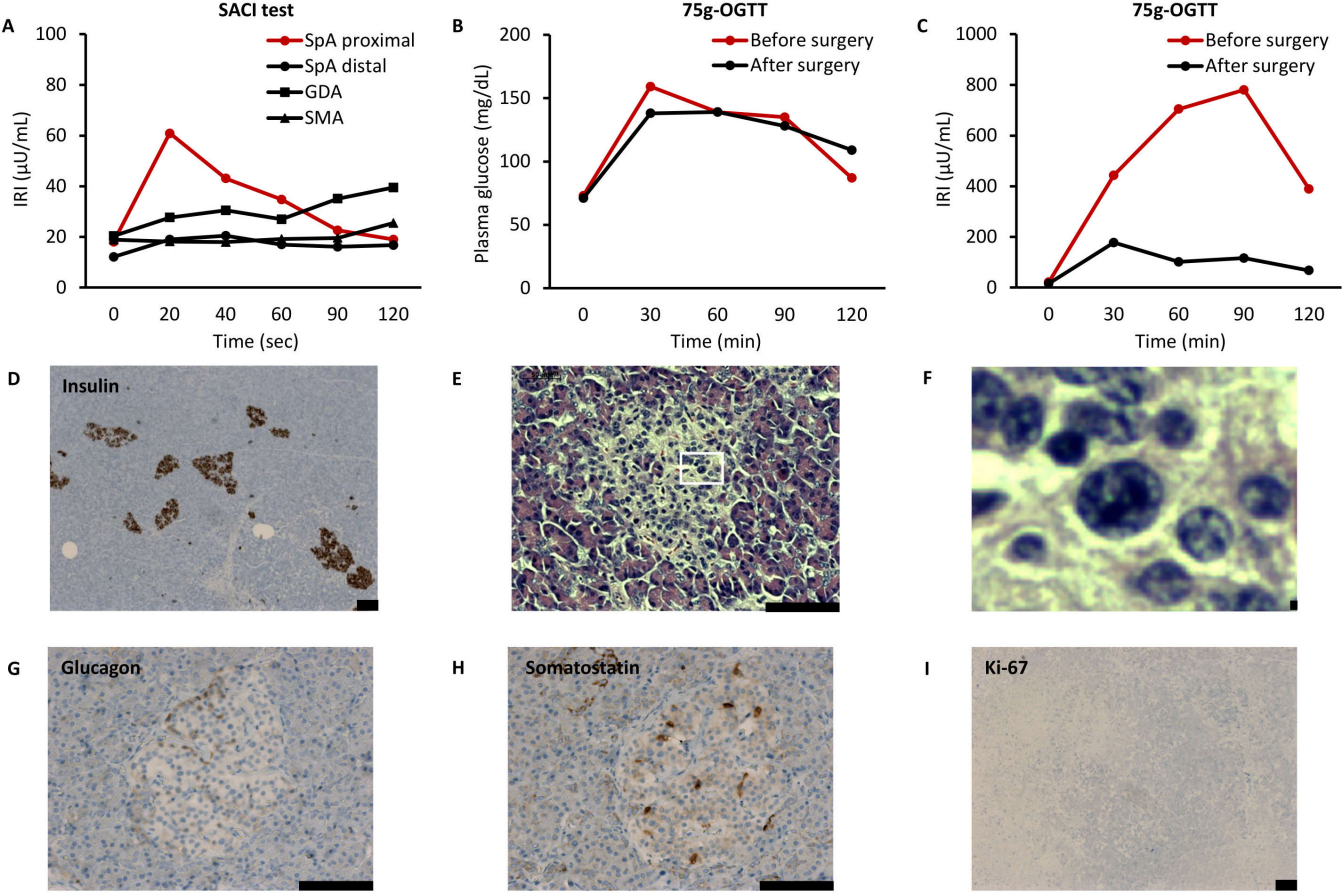
Results of clinical examinations in the present case. **(A)** Selective arterial calcium injection (SACI) test. IRI, immunoreactive insulin; SpA, splenic artery; GDA, gastroduodenal artery; and SMA, superior mesenteric artery. **(B, C)** 75-g oral glucose tolerance test (OGTT). Vertical axes show plasma glucose **(B)** and immunoreactive insulin (IRI) **(C)**. **(D–I)** Histological examinations of resected pancreas. Immunohistochemistry of insulin (D: magnification, ×50), hematoxylin & eosin staining (**E**: magnification, ×200, **F**: ×10 expansion of **E**), immunohistochemistry of glucagon (**G**: magnification, ×200), immunohistochemistry of somatostatin (**H**: magnification, ×200), and immunohistochemistry of Ki-67 (**I**: magnification, ×50). Scale bars indicate 100 µm.

His past medical history was notable for mental retardation and surgery for squinting, but he had no significant family history including his two younger brothers. His body mass index was calculated as 25.4 kg/m^2^ based on a height of 159.1 cm and a body weight of 64.3 kg. The final height of his father was 170 cm and that of his mother was 160 cm. Blood test results at 25 years of age are listed in **Supplementary Table 1**. We diagnosed him with HH on the basis of the following results after 66-hour fasting: low plasma glucose (44 mg/dL) and unsuppressed IRI (6.2 µU/mL) (**Supplementary Table 2**) (8). We performed contrast-enhanced CT, MRI, and EUS again, which did not detect any pancreatic tumors. Selective arterial calcium injection identified an increase in IRI when calcium gluconate was injected into the proximal portion of the splenic artery: the IRI before the injection was 18.1 µU/mL and the peak IRI was 60.9 µU/mL at 20 s after the injection (**Figure 1A**). We performed a 75-g oral glucose tolerance test (OGTT) and found a striking elevation of IRI: the IRI before intake was 22.1 µU/mL, with a plasma glucose level of 73 mg/dL, and the peak IRI was 780.3 µU/mL after 90 min (**Figures 1B, C**).

The fact that his fasting test results became abnormal indicated the progressive nature of his HH. Furthermore, he had become symptomatic, as seen by his increased body weight and elevated transaminases: aspartate aminotransferase (AST) was 101 U/L and alanine aminotransferase (ALT) was 234 U/L (**Supplementary Table 1**). CT imaging revealed the presence of fatty liver, with a CT value of 36.8 Hounsfield Unit (HU). We prescribed 75 mg of diazoxide in three divided doses. His increased appetite subsided, and his body weight decreased to 60.0 kg 6 weeks after initiating diazoxide. In addition, AST decreased to 47 IU/L and ALT decreased to 78 IU/L. Thus, improvement of his HH appeared to be significant. We decided to perform laparoscopic distal pancreatectomy because we considered this could cure his HH regardless of whether it was caused by nesidioblastosis or occult insulinoma.

As expected, HH improved postoperatively, with a 72-h fasting test resulting in a plasma glucose level of 58 mg/dL and an IRI of 5.2 µU/mL (**Supplementary Table 2**). The IRI was markedly decreased during a repeat 75 g-OGTT: before intake the IRI was 16.0 µU/mL, with a plasma glucose level of 71 mg/dL, and the peak IRI was 177.8 µU/mL after 30 min (**Figures 1B, C**). In addition, the area under the curve of the IRI during the 75-g OGTT was 65.7% lower than that before surgery, which almost corresponded to the extent of the resected pancreas. Improvement of HH was maintained even 2 years after surgery: in a 75 g-OGTT, before intake the IRI was 7.4 µU/mL, with a plasma glucose level of 64 mg/dL, and the peak IRI was 121.5 µU/mL after 60 min. His body weight decreased to 51.5kg, AST decreased to 25 IU/L, and ALT decreased to 38 IU/L. Furthermore, CT imaging revealed improvement in fatty liver, with an elevated CT value of 50.4 HU.

Histological examinations of the resected pancreas showed no neoplastic lesions or any invasion of pancreatic islets into the pancreatic ducts (**Figure 1D**). There were no histological findings of nesidioblastosis that are characteristically seen in newborns, such as diffuse or disseminated proliferation of islet cells (15). Meanwhile, some β cells exhibited enlarged hyperchromatic nuclei and clear cytoplasm (**Figures 1E, F**). The islets contained glucagon-positive cells and somatostatin-positive cells with normal spatial distribution (**Figures 1G, H**). There seemed to be an absence of proliferative activity, as almost no islet cells were stained with Ki-67 (**Figure 1I**). These findings were compatible with the pathological criteria of adult nesidioblastosis (11). Thus, we concluded that the patient’s HH was caused by nesidioblastosis.

### Genetic analysis

**Figure 2:**
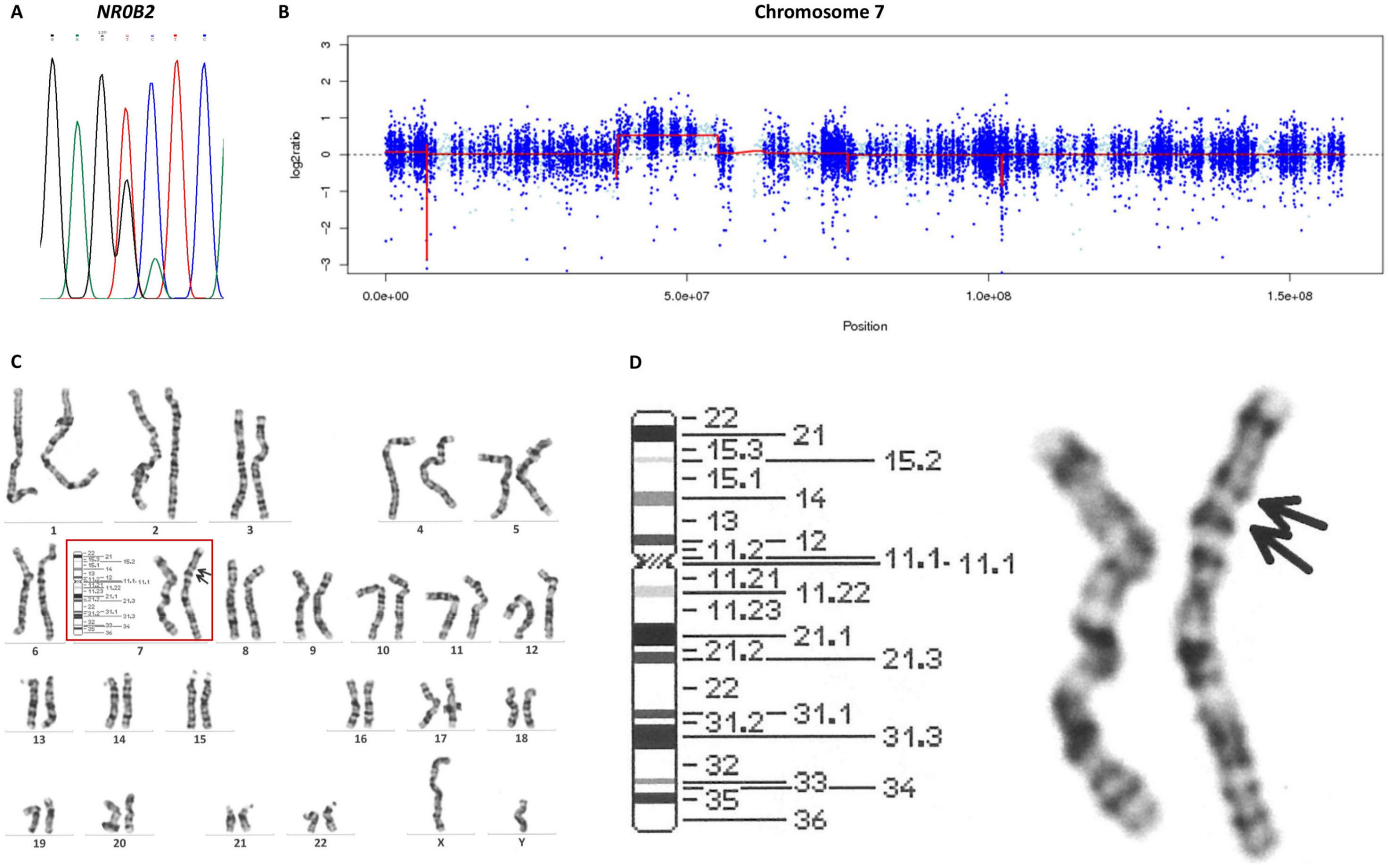
Genetic analyses in the present case. **(A)** Sanger sequencing of the *NR0B2* gene revealed the heterogenous c.583G>T variant. **(B)** A copy number analysis with EXCAVATOR2 suggested duplication of chromosome 7p11.2–p14.1. **(C)** Images of all chromosomes in G-banding karyotyping. **(D)** Chromosome 7 in G-banding karyotyping revealed dup(7)(p11.2p14).

The juvenile onset of his nesidioblastosis and its slowly progressive nature suggested a congenital etiology. Therefore, we initiated genetic examinations at Kyoto University. After obtaining informed consent from the patient, his genomic DNA was extracted from peripheral blood leukocytes using a NucleoSpin Blood kit (Macherey-Nagel, Düren, Germany). We outsourced whole-exome sequencing (WES) to Macrogen Japan (Tokyo, Japan). The sequencing library was generated using a SureSelect Human All ExonV6 kit (Agilent Technologies, Santa Clara, CA) and was sequenced using the NovaSeq 6000 platform (Illumina, Inc., San Diego, CA). No pathogenic variants were called in the following genes known to be associated with HH secondary to CHI: *ABCC8*, *KCNJ11*, *GCK*, *GLUD-1*, *HADH1*, *SLC16A1*, *HNF1A*, *HNF4A*, *UCP2*, *HK1*, *PGM1*, and *PMM2*. Interestingly, we found a heterozygous variant in the *NR0B2* gene: c.583G>T p.Ala195Ser. Specific amplifications of this gene were performed using a polymerase chain reaction (PCR) technique with the following primers: *NR0B2* forward, 5′-CAGATCTTGGGCCAGTCTTG-3′; *NR0B2* reverse, 5′-CTCCAGGAGCATTGGGTCAC-3′. Sanger sequencing of PCR products, conducted by Macrogen Japan, confirmed this *NR0B2* variant (**Figure 2A**). His mother did not carry this variant. We could not examine his father’s genotype because he was uncontactable. Likewise, we outsourced WES of the resected pancreas specimen to Macrogen Japan, which revealed that no pathogenic variants were called in the HH-associated genes: *ABCC8*, *KCNJ11*, *GCK*, *GLUD-1*, *HADH1*, *SLC16A1*, *HNF1A*, *HNF4A*, *UCP2*, *HK1*, *PGM1*, and *PMM2*.

The Initiative on Rare and Undiagnosed Diseases (IRUD), a unified all-Japan diagnostic and research program for rare and undiagnosed diseases, also conducted WES (16). After obtaining informed consent from the patient and the patient’s mother, their genomic DNA was extracted from peripheral blood leukocytes using the standard phenol extraction protocol. All the exons were captured using a SureSelect XT Human All Exon V6 kit (Agilent Technologies); then, exome analyses were performed using the NovaSeq 6000 platform (Illumina, Inc.). EXCAVATOR2 software, which screens for copy number variants using aligned exome data in bam format (17, 18), revealed that the patient had a heterozygous duplication of approximately 18 Mbp in chromosome 7p11.2–p14.1 (location: 38,530,605–55,137,273) (**Figure 2B**) and his mother did not. G-banding karyotyping of peripheral lymphocytes by LSI Medience Corporation (Tokyo, Japan) confirmed this abnormal mosaic karyotype: mos 46,XY,dup(7)(p11.2p14) (12)/46,XY (18) (**Figures 2C, D**). To examine whether the patient’s chromosome with the duplication was inherited from his mother, we analyzed single nucleotide polymorphisms (SNPs) located near a *GRB10* gene on chromosome 7p11.2–p14.1, which were heterozygous in the patient and homozygous in the mother. If there were no duplication in these regions, the read ratio would converge around 50%. However, the read ratios of the maternal SNPs in the patient ranged from 63.3–71.9%, suggesting that the chromosome with the duplication is maternally derived (**Supplementary Table 3**).

We suspected with SRS-like phenotype from this mosaic karyotype because duplication of regions including 7p11.2–p14 was reported to cause SRS-like phenotype (13, 14, 19, 20). No point mutations were called from WES data in the following genes associated with SRS: *H19*, *IGF2*, *CDKN1C*, *GRB10*, and *MEST* (1). We reviewed the patient’s clinical histories and physical findings. His birth weight was 2410 g (−2.06 SD), height was 47.0 cm (−1.28 SD), and head circumference was 29.0 cm (−3.25 SD), indicating that he was small for his gestational age (39 weeks and 6 days). His growth curves showed that he experienced postnatal growth failure affecting both his height and body weight with feeding difficulty not requiring up to tube feeding (**Supplementary Figure 1A**). There was no macrocephaly, and in fact his head circumference was rather small (**Supplementary Figure 1B**). There was also no body asymmetry. As a result, he met two NH-CSS criteria. Additional relevant features included bilateral fifth finger clinodactyly (**Supplementary Figures 2A, B**) and intellectual disability. Regarding etiology of his intellectual disability, neither hypoglycemia nor convulsions were noted in infancy. In addition, global developmental delay was reported in 34% cases of SRS, and upd(7)mat was more likely to present with global developmental delay than 11p15 LOM (4). Since pathophysiology of dup(7) is speculated to be similar to that of upd(7)mat, we believe that his intellectual disability originated from SRS-like phenotype. However, occult continuous hypoglycemia remains a potential contributing factor. Thus, we determined that he had SRS-like phenotype on the basis of clinical and genetic features.

## Discussion

Here we report an adult male patient with SRS-like phenotype and concurrent HH caused by nesidioblastosis. Genetic analyses identified the pathogenic background as the heterozygous *NR0B2* Ala195Ser variant and mosaic dup(7)(p11.2p14). A relationship between these findings must be considered because both nesidioblastosis and SRS-like phenotype are rare.

The *NR0B2* gene encodes the so-called small heterodimer partner, an orphan member of the mammalian nuclear receptor superfamily. Its endogenous ligands have not been identified, though its regulatory function is mediated by protein-protein interactions with other nuclear receptors and transcription factors (21). *NR0B2* acts as a negative regulator of insulin secretion by islets and regulates the synthesis of bile acid and the metabolism of cholesterol and lipid (21, 22). Nishigori et al. reported that patients with early-onset diabetes who harbored *NR0B2* variants presented with hyperinsulinemia and obesity, and that mutant proteins, including Ala195Ser, exhibited weaker suppression of the transactivation of HNF-4α, a positive modulator of insulin secretion (23). Thus, the pathogenicity of *NR0B2* Ala195Ser variant is classified to “likely pathogenic (PS3 and PM6)” according to the variant classification guidelines from the American College of Medical Genetics and Genomics/Association for Molecular Pathology (ACMG/AMP) (24).

The frequency of the *NR0B2* Ala195Ser variant in the Genome Aggregation Database (gnomAD, v4.0.0) is extremely low, at only 7.4×10^-5^. However, its frequency in gnomAD is not as low in East Asian individuals (1.4×10^-3^, 58/41274), and it is not seen in non-East Asian people (0, 0/739670). Furthermore, in 54KJPN, a Japanese whole-genome reference panel, the frequency of this variant in the Japanese population is 4 × 10^-3^ (429/108604). Considering that HH due to adult nesidioblastosis is extremely rare, the *NR0B2* Ala195Ser variant is unlikely to cause HH alone. It should be noted that this variant was unlikely to be related to the etiology of his HH, though the true association could not be determined.

In this study, we detected dup(7)(p11.2p14) and observed that the patient had SRS-like phenotype. His mother did not have dup(7)(p11.2p14), but this did not conflict with his genotype. In this case, the duplication site was presumed to be maternally oriented and not silenced, as the SRS-like phenotype was present and the analysis of SNPs near the *GRB10* gene, one of the candidate gene for SRS, also suggested maternal origin. It is speculated that duplication of maternal origin occurred due to somatic mosaicism. The molecular mechanism of SRS-like phenotype due to dup(7) is considered to be similar to that associated with upd(7)mat. We performed a literature review of SRS-like phenotype due to dup(7), and list the identified studies in **Table 1** (13, 14, 19, 20). The NH-CSS score in our case was only two, which was not high, and in six previous cases of SRS-like phenotype caused by dup(7), it was also low, ranging from 0 to 2. SRS-like phenotype due to dup(7) seems to present with mild phenotypes as SRS due to upd(7)mat presents with fewer typical clinical features than 11p15 LOM (3, 4). All reported cases of SRS-like phenotype due to dup(7) included *GCK* gene in the duplication region (13, 14, 19, 20). Interestingly, the patients in one previous case had hypoglycemia (13), and one also had hyperinsulinemia (14). Other reports did not mention hypoglycemia or nesidioblastosis.

**Table 1:** A literature review of cases with SRS-like phenotype due to duplication of chromosome 7 (dup7).

Patient	Sex	Age (years)	Genotype	NH-CSS	Hypoglycemia	References
1	Female	6	dup(7)(p12.1p13)	0/6	NA	(20)
2	Female	NA	dup(7)(p12.1p13)	0/6	NA	(20)
3	Female	5	dup(7)(p11.2p13)	2/6	+	(13)
4	Male	4	dup(7)(p11.2p13)	2/6	NA	(19)
5	Female	48	dup(7)(p11.2p13)	1/6	NA	(19)
6	Female	17	dup(7)(p11.2p13)	2/6	Hyperinsulinemia alone	(14)
7	Male	25	dup(7)(p11.2p14)	2/6	+	Present case

SRS, Silver-Russel syndrome; NH-CSS, Netchine-Harbison clinical scoring system; NA, not available.

+, presence of hypoglycemia.

Wakeling et al. reported the prevalence of hypoglycemia in SRS according to karyotype (4): 24% for 11p15 LOM and 29% for upd(7)mat. Thus, hypoglycemia is a frequent complication of SRS but its etiology is not well clarified. Azcona et al. examined 24 patients with both SRS and hypoglycemia, and concluded that the most likely causes of hypoglycemia were accelerated starvation and/or growth hormone insufficiency (6). They found no hormonal abnormalities except for growth hormone insufficiency, but their study lacked information on karyotypes (6). Thus it remains unclear if the etiology of hypoglycemia in SRS differs between patients with 11p15LOM, upd(7)mat, or dup(7).

**Figure 3:**
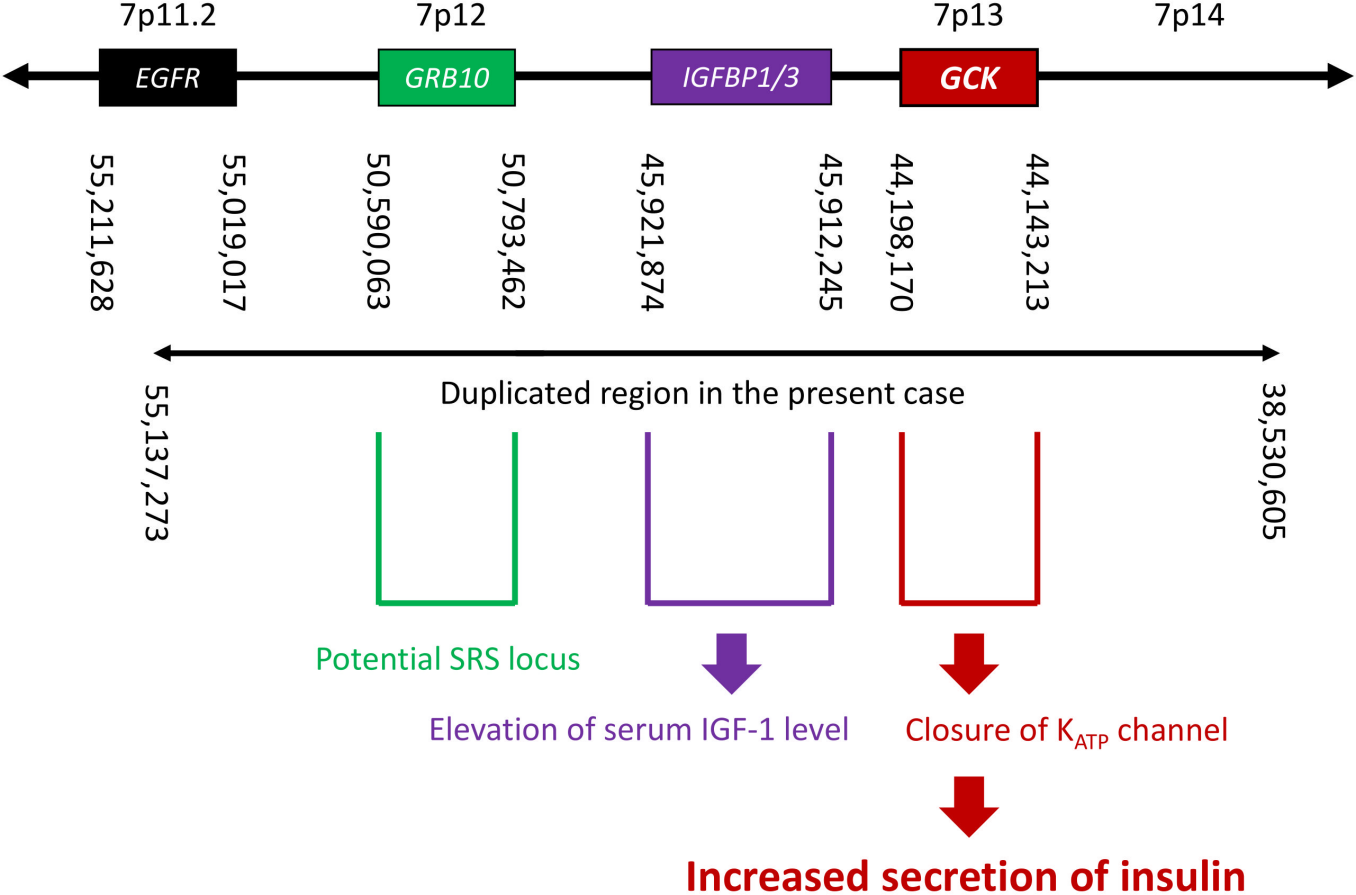
A schema of the genome around 7p11.2–p14, showing the locations of nearby genes, breakpoints suggested by EXCAVATOR2 analysis, and estimated correlations between genotype and phenotype. SRS, Silver-Russell syndrome.

It is informative that in the present case, the cause of hypoglycemia was confirmed to be nesidioblastosis. Furthermore, his hypoglycemia was not noted during childhood, which is atypical for SRS and does not suggest involvement of starvation and/or growth hormone insufficiency. To identify a potential association between nesidioblastosis and dup(7), we searched for genes located in the duplicated region and found the *GCK* gene at 7p13 (**Figure 3**). The *GCK* gene encodes glucokinase, which controls glycogen synthesis in the liver and acts as a primary glucose sensor that regulates insulin secretion in pancreatic islets (25). In pancreatic β cells, glucokinase catalyzes the phosphorylation of glucose to glucose-6-phosphate in the glycolytic pathway; this results in an increase in the ATP: ADP ratio, which in turn leads to the closing of K_ATP_ channels and the promotion of insulin secretion (26, 27). Importantly, heterozygous activating mutations in the *GCK* gene cause mild HH (28). Furthermore, previous reports have shown hypoglycemia and increased *GCK* activity in transgenic mice that have one additional copy of the *GCK* gene though the difference of plasma insulin level was not detected (29, 30); this genetic background is similar to that our case, in which the *GCK* gene itself was duplicated but not mutated.

In our patient, dup(7) was a mosaic, detected in only 12 of 30 (40%) peripheral blood mononuclear cells. Islet β cells are heterogenous, and Hub cells with high *GCK* expression preferentially respond to insulin secretory stimuli, which results in the propagation of signals throughout the islet via inter-β-cell gap junctions (31). Furthermore, the activation of *GCK* in a minority of β cells is sufficient to change the glucose threshold for insulin secretion in the entire islet and thereby cause HH (32). Taken together, we consider that in our patient, duplication of the *GCK* gene caused HH due to nesidioblastosis, even though he had mosaic duplications. However, a limitation remains in that we could not obtain direct evidence confirming that the patient’s pancreas has dup(7). Although we analyzed using EXCAVATOR2 software and quantitative PCR, these analyses were unsuccessful due to the low quality of the genomic DNA extracted from the pancreatic specimen, which had deteriorated over time.

Given the pathophysiology in our patient, it is reasonable to assume that the *GCK* gene could also mediate hypoglycemia in SRS due to upd(7)mat. Hypoglycemia in SRS due to 11p15 LOM seems unrelated to the *GCK* gene, as demonstrated by a previous study (6). The relationship between elevated transaminases and duplication of the *GCK* gene remains unclear. The duplication of the *IGFBP1/3* gene can increase their expression (**Figure 3**). When IGFBP3 binds to IGF1, bound IGF1 is inactivated, leading to a decrease in free IGF1, the active form (33). The binding complex extends the half-life of IGF1 in circulation. Total IGF1 increases due to this prolonged half-life, while free IGF1 may be reduced, potentially contributing to his short stature.

## Conclusion

Our patient presented with SRS-like phenotype complicated with HH due to nesidioblastosis. Genetic analyses revealed that his SRS-like phenotype was caused by mosaic dup(7)(p11.2p14). While the concomitant heterozygous *NR0B2* Ala195Ser variant might have contributed to some extent, duplication of the *GCK* gene along with dup(7)(p11.2p14) was the most likely cause of his nesidioblastosis. Although further studies are required to confirm the etiology of hypoglycemia in SRS-like phenotype due to dup(7) and SRS due to upd(7)mat, duplication of the *GCK* gene is suggested as a novel cause of nesidioblastosis.

## Data Availability

The original contributions presented in the study are included in the article/**Supplementary Material**. Further inquiries can be directed to the corresponding author.

## References

[1] Wakeling EL Brioude F Lokulo-Sodipe O O’Connell SM Salem J Bliek J . Diagnosis and management of Silver–Russell syndrome: first international consensus statement. Nat Rev Endocrinol. (2017) 13:105–24. doi: 10.1038/nrendo.2016.138 27585961

[2] Azzi S Salem J Thibaud N Chantot-Bastaraud S Lieber E Netchine I . Original article: A prospective study validating a clinical scoring system and demonstrating phenotypical-genotypical correlations in Silver-Russell syndrome. J Med Genet. (2015) 52:446–53. doi: 10.1136/jmedgenet-2014-102979 PMC4501172 25951829

[3] Bartholdi D Krajewska-Walasek M Õunap K Gaspar H Chrzanowska KH Ilyana H . Epigenetic mutations of the imprinted IGF2-H19 domain in Silver-Russell syndrome (SRS): results from a large cohort of patients with SRS and SRS-like phenotypes. J Med Genet. (2009) 46:192–7. doi: 10.1136/jmg.2008.061820 19066168

[4] Wakeling EL Abu Amero S Alders M Bliek J Forsythe E Kumar S . Epigenotype–phenotype correlations in Silver–Russell syndrome. J Med Genet. (2010) 47:760–8. doi: 10.1136/jmg.2010.079111 PMC2976034 20685669

[5] Netchine I Rossignol S Dufourg MN Azzi S Rousseau A Perin L . 11p15 imprinting center region 1 loss of methylation is a common and specific cause of typical russell-silver syndrome: clinical scoring system and epigenetic-phenotypic correlations. J Cli Endocrinol Metab. (2007) 92:3148–54. doi: 10.1210/jc.2007-0354 17504900

[6] Azcona C Stanhope R . Hypoglycaemia and russell-silver syndrome. J Pediatr Endocrinol Metab. (2005) 18:663–70. doi: 10.1515/JPEM.2005.18.7.663 16128243

[7] Cryer PE Axelrod L Grossman AB Heller SR Montori VM Seaquist ER . Evaluation and management of adult hypoglycemic disorders: an endocrine society clinical practice guideline. J Clin Endocrinol Metab. (2009) 94:709–28. doi: 10.1210/jc.2008-1410 19088155

[8] Service FJ . Hypoglycemic disorders. N Engl J Med. (1995) 332:1144–52. doi: 10.1056/NEJM199504273321707 7700289

[9] Yamada Y Kitayama K Oyachi M Higuchi S Kawakita R Kanamori Y . Nationwide survey of endogenous hyperinsulinemic hypoglycemia in Japan (2017-2018): Congenital hyperinsulinism, insulinoma, non-insulinoma pancreatogenous hypoglycemia syndrome and insulin autoimmune syndrome (Hirata’s disease). J Diabetes Investig. (2020) 11:554–63. doi: 10.1111/jdi.13180 PMC7232294 31742894

[10] Dieterle MP Husari A Prozmann SN Wiethoff H Stenzinger A Röhrich M . Diffuse, adult-onset nesidioblastosis/non-insulinoma pancreatogenous hypoglycemia syndrome (NIPHS): review of the literature of a rare cause of hyperinsulinemic hypoglycemia. Biomedicines. (2023) 11:1732. doi: 10.3390/biomedicines11061732 37371827 PMC10296556

[11] Anlauf M Wieben D Perren A Sipos B Komminoth P Raffel A . Persistent hyperinsulinemic hypoglycemia in 15 adults with diffuse nesidioblastosis: Diagnostic criteria, incidence, and characterization of β-cell changes. Am J Surg Pathol. (2005) 29:524–33. doi: 10.1097/01.pas.0000151617.14598.ae 15767809

[12] Demirbilek H Hussain K . Congenital hyperinsulinism: diagnosis and treatment update. J Clin Res Pediatr Endocrinol. (2017) 9:69–87. doi: 10.4274/jcrpe.2017.S007 29280746 PMC5790328

[13] Monk D Wakeling EL Proud V Hitchins M Abu-Amero SN Stanier P . Duplication of 7p11.2-p13, including GRB10, in silver-russell syndrome. Am J Hum Genet. (2000) 66:36–46. doi: 10.1086/302717 10631135 PMC1288348

[14] Catusi I Bonati MT Mainini E Russo S Orlandini E Larizza L . Recombinant chromosome 7 driven by maternal chromosome 7 pericentric inversion in a girl with features of silver-russell syndrome. Int J Mol Sci. (2020) 21:1–11. doi: 10.3390/ijms21228487 PMC7698152 33187293

[15] Yakovac WC Baker L Hummeler K . Beta cell nesidioblastosis in idiopathic hypoglycemia of infancy. J Pediatr. (1971) 79:226–31. doi: 10.1016/S0022-3476(71)80105-1 4104455

[16] Takahashi Y Date H Oi H Adachi T Imanishi N Kimura E . Six years’ accomplishment of the Initiative on Rare and Undiagnosed Diseases: nationwide project in Japan to discover causes, mechanisms, and cures. J Hum Genet. (2022) 67:505–13. doi: 10.1038/s10038-022-01025-0 PMC9402437 35318459

[17] D’Aurizio R Pippucci T Tattini L Giusti B Pellegrini M Magi A . Enhanced copy number variants detection from whole-exome sequencing data using EXCAVATOR2. Nucleic Acids Res. (2016) 44:e154. doi: 10.1093/nar/gkw695 27507884 PMC5175347

[18] Suzuki H Yamada M Uehara T Takenouchi T Kosaki K . Parallel detection of single nucleotide variants and copy number variants with exome analysis: Validation in a cohort of 700 undiagnosed patients. Am J Med Genet A. (2020) 182:2529–32. doi: 10.1002/ajmg.a.v182.11 PMC7689761 32779332

[19] Monk D Bentley L Hitchins M Myler RA Clayton-Smith J Ismail S . Chromosome 7p disruptions in Silver Russell syndrome: delineating an imprinted candidate gene region. Hum Genet. (2002) 111:376–87. doi: 10.1007/s00439-002-0777-4 12384779

[20] Joyce CA Sharp A Walker JM Bullman H Temple IK . Duplication of 7p12.1-p13, including GRB10 and IGFBP1, in a mother and daughter with features of Silver-Russell syndrome. Hum Genet. (1999) 105:273–80. doi: 10.1007/s004390051101 10987657

[21] Zhang Y Hagedorn CH Wang L . Role of nuclear receptor SHP in metabolism and cancer. Biochim Biophys Acta. (2011) 1812:893–908. doi: 10.1016/j.bbadis.2010.10.006 20970497 PMC3043166

[22] Chanda D Park JH Choi HS . Molecular basis of endocrine regulation by orphan nuclear receptor Small Heterodimer Partner. Endocr J. (2008) 55:253–68. doi: 10.1507/endocrj.K07E-103 17984569

[23] Nishigori H Tomura H Tonooka N Kanamori M Yamada S Sho K . Mutations in the small heterodimer partner gene are associated with mild obesity in Japanese subjects. Proc Natl Acad Sci U.S.A. (2001) 98:575–80. doi: 10.1073/pnas.98.2.575 PMC14629 11136233

[24] Richards S Aziz N Bale S Bick D Das S Gastier-Foster J . Standards and guidelines for the interpretation of sequence variants: A joint consensus recommendation of the american college of medical genetics and genomics and the association for molecular pathology. Genet Med. (2015) 17:405–24. doi: 10.1038/gim.2015.30 PMC4544753 25741868

[25] Sternisha SM Miller BG . Molecular and cellular regulation of human glucokinase. ArchBiochem Biophys. (2019) 663:199–213. doi: 10.1016/j.abb.2019.01.011 PMC6377845 30641049

[26] Senniappan S Arya VB Hussain K . The molecular mechanisms, diagnosis and management of congenital hyperinsulinism. Indian J Endocrinol Metab. (2013) 17:19–30. doi: 10.4103/2230-8210.107822 23776849 PMC3659902

[27] Rahman SA Nessa A Hussain K . Molecular mechanisms of congenital hyperinsulinism. J Mol Endocrinol. (2015) 54:R119–129. doi: 10.1530/JME-15-0016 25733449

[28] Osbak KK Colclough K Saint-Martin C Beer NL Bellanné-Chantelot C Ellard S . Update on mutations in glucokinase (GCK), which cause maturity-onset diabetes of the young, permanent neonatal diabetes, and hyperinsulinemic hypoglycemia. Hum Mutat. (2009) 30:1512–26. doi: 10.1002/humu.v30:11 19790256

[29] Niswender KD Postic C Jetton TL Bennett BD Piston DW Efrat S . Cell-specific expression and regulation of a glucokinase gene locus transgene. J Biol Chem. (1997) 272:22564–9. doi: 10.1074/jbc.272.36.22564 9278410

[30] Niswender KD Shiota M Postic C Cherrington AD Magnuson MA . Effects of increased glucokinase gene copy number on glucose homeostasis and hepatic glucose metabolism. J Biol Chem. (1997) 272:22570–5. doi: 10.1074/jbc.272.36.22570 9278411

[31] Johnston NR Mitchell RK Haythorne E Pessoa MP Semplici F Ferrer J . Beta cell hubs dictate pancreatic islet responses to glucose. Cell Metab. (2016) 24:389–401. doi: 10.1016/j.cmet.2016.06.020 27452146 PMC5031557

[32] Chen KH Doliba N May CL Roman J Ustione A Tembo T . Genetic activation of glucokinase in a minority of pancreatic beta cells causes hypoglycemia in mice. Life Sci. (2022) 309:120952. doi: 10.1016/j.lfs.2022.120952 36100080 PMC10312065

[33] Blum WF Alherbish A Alsagheir A Awwa AE Kaplan W Koledova E . The growth hormone-insulin-like growth factor-I axis in the diagnosis and treatment of growth disorders. Endocr Connect. (2018) 7:212–22. doi: 10.1530/EC-18-0099 PMC5987361 29724795

