# Supplementary figures and images for: Case report: Duplication of the GCK gene is a novel cause of nesidioblastosis: evidence from a case with Silver-Russell syndrome-like phenotype related to chromosome 7
Source: Front Endocrinol (Lausanne). 2024 Dec 10;15:1431547. doi: 10.3389/fendo.2024.1431547 (PMC11666348; doi:10.3389/fendo.2024.1431547)

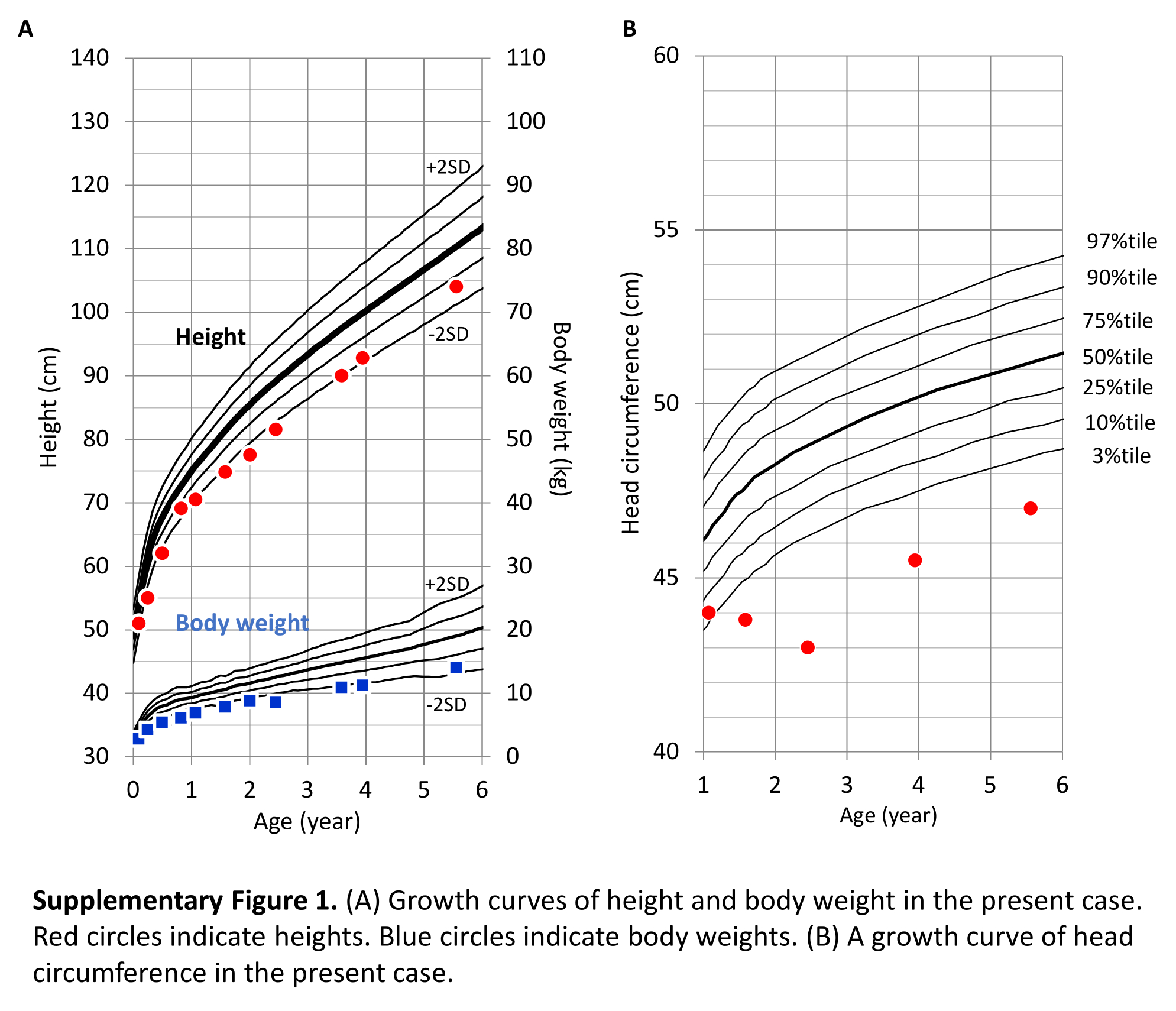

Supplement: Supplementary file 1 [file Image1.jpg]

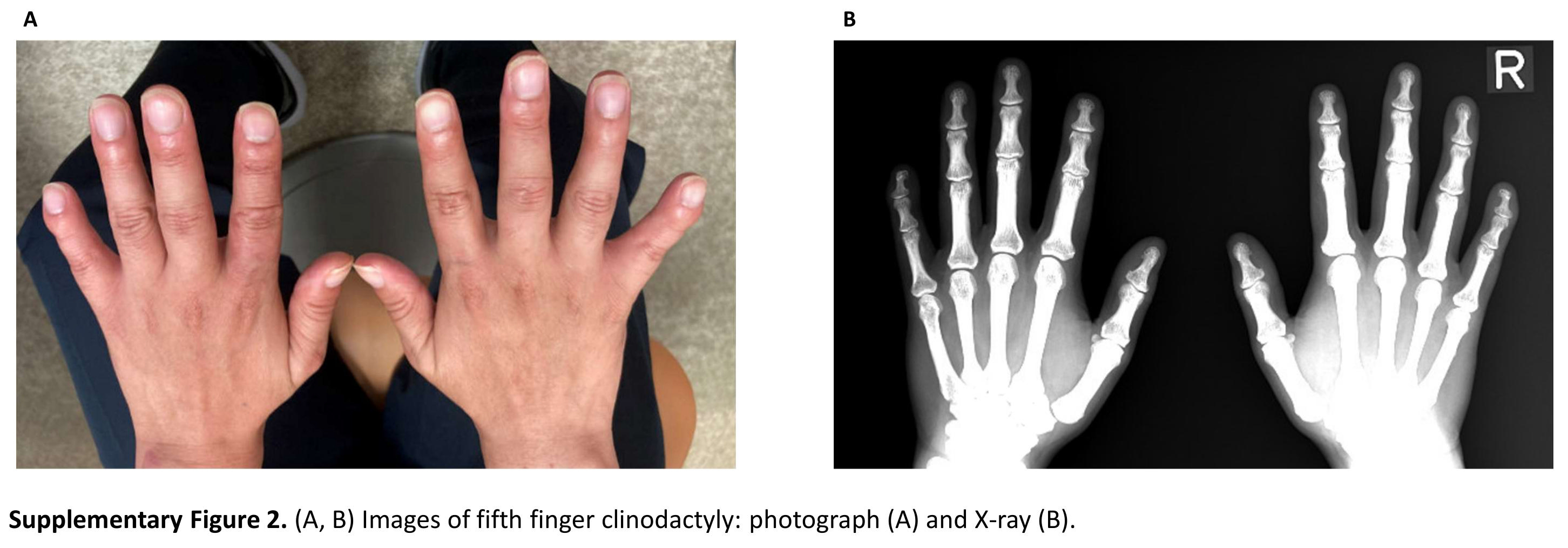

Supplement: Supplementary file 2 [file Image2.jpg]
