# Supplementary material for: Case report: Duplication of the GCK gene is a novel cause of nesidioblastosis: evidence from a case with Silver-Russell syndrome-like phenotype related to chromosome 7
Source: Front Endocrinol (Lausanne). 2024 Dec 10;15:1431547. doi: 10.3389/fendo.2024.1431547 (PMC11666348; doi:10.3389/fendo.2024.1431547)
Supplement: Supplementary file 3 [file Table1.docx]

**Supplementary Table 1.** **Blood test results at 25 years of age**

|  | Result | Reference range |
| --- | --- | --- |
| White blood cells (/μL) | 3590 | 3300–8600 |
| Hemoglobin (g/dL) | 16.4 | 13.5–16.7 |
| Platelets (10^4^/μL) | 23.9 | 15.9–38.3 |
| Total protein (g/dL) | 7.4 | 6.3–8.1 |
| Albumin (g/dL) | 4.3 | 3.9–5.1 |
| Aspartate aminotransferase (U/L) | 101 | 12–30 |
| Alanine aminotransferase (U/L) | 234 | 10–42 |
| Lactate dehydrogenase (U/L) | 237 | 124–226 |
| Alkaline phosphatase (U/L) | 75 | 40–126 |
| γ-glutamyl transpeptidase (U/L) | 60 | 9–54 |
| Total bilirubin (mg/dL) | 1.1 | 0.3–1.3 |
| Amylase (U/L) | 131 | 45–140 |
| Creatinine (mg/dL) | 0.67 | 0.65–1.06 |
| Urea nitrogen (mg/dL) | 18 | 8–22 |
| Uric acid (mg/dL) | 9.1 | 3.8–7.0 |
| Creatine kinase (U/L) | 218 | 61–257 |
| Total cholesterol (mg/dL) | 149 | 140–220 |
| High-density lipoprotein cholesterol (mg/dL) | 51 | 40–91 |
| Low-density lipoprotein cholesterol (mg/dL) | 74 | 62–140 |
| Triglyceride (mg/dL) | 125 | 40–150 |
| Sodium (mmol/L) | 138 | 137–144 |
| Potassium (mmol/L) | 4.3 | 3.6–4.8 |
| Chlorine (mmol/L) | 101 | 101–108 |
| Calcium (mg/dL) | 9.4 | 8.7–10.1 |
| Phosphate (mg/dL) | 3.9 | 2.6–4.5 |
| Hemoglobin A1c (%) | 4.6 | 4.6–6.2 |
| Anti-insulin antibody | Negative | Negative |
| Thyroid stimulating hormone (µIU/mL) | 1.290 | 0.5–5.0 |
| Free thyroxine (ng/dL) | 1.370 | 0.88–1.62 |
| Adrenocorticotropic hormone (pg/mL) | 39.4 | 7.2–63.3 |
| Cortisol (µg/dL) | 16.1 | 5–15 |
| Growth hormone (ng/mL) | 0.35 | <2.10 |
| Insulin-like growth factor-1 (ng/mL) | 430 | 125–337 |
