# Supplementary material for: Case report: Duplication of the GCK gene is a novel cause of nesidioblastosis: evidence from a case with Silver-Russell syndrome-like phenotype related to chromosome 7
Source: Front Endocrinol (Lausanne). 2024 Dec 10;15:1431547. doi: 10.3389/fendo.2024.1431547 (PMC11666348; doi:10.3389/fendo.2024.1431547)
Supplement: Supplementary file 4 [file Table2.docx]

**Supplementary Table 2. Fasting test before and after pancreatectomy**

|  | Before | After | Reference |
| --- | --- | --- | --- |
| Glucose (mg/dL) | 44 | 58 | < 45 |
| IRI (µU/mL) | 6.2 | 5.2 | ≥ 6 |
| C-peptide (nmol/L) | 0.56 | 0.39 | ≥ 0.2 |
| Proinsulin (pmol/L) | 7.0 | 1.42 | ≥ 5 |
| Betahydroxybutyrate (mmol/L) | 2.17 | 2.13 | ≤ 2.7 |

The blood sample before pancreatectomy was collected after fasting for 66 h, and that after pancreatectomy was collected after fasting for 72 h. Reference values are from Ref. 8. IRI, immunoreactive insulin.
