# Supplementary material for: Case report: Duplication of the GCK gene is a novel cause of nesidioblastosis: evidence from a case with Silver-Russell syndrome-like phenotype related to chromosome 7
Source: Front Endocrinol (Lausanne). 2024 Dec 10;15:1431547. doi: 10.3389/fendo.2024.1431547 (PMC11666348; doi:10.3389/fendo.2024.1431547)
Supplement: Supplementary file 5 [file Table3.docx]

**Supplementary Table 3.** **An analysis of single nucleotide polymorphisms located near the *GRB10* gene**

| Chromosome  Position | 7  50173777 | 7  51096036 | 7  53103371 | 7  53103382 | 7  53104078 |
| --- | --- | --- | --- | --- | --- |
| Gene | *C7orf72* | *COBL* | *POM121L12* | *POM121L12* | *POM121L12* |
| Reference allele | A | G | G | G | G |
| Alternative allele | G | C | T | T | A |
| Read count (Mother) |  |  |  |  |  |
| Reference allele | 0 | 130 | 54 | 61 | 201 |
| Alternative allele | 26 | 0 | 1 | 0 | 1 |
| Read count (Patient) |  |  |  |  |  |
| Reference allele | 18 | 246 | 57 | 65 | 285 |
| Alternative allele | 31 | 96 | 23 | 28 | 143 |
| Maternal ratio (%) | 63.3 | 71.9 | 71.3 | 69.9 | 66.6 |

In the region from 49,000,000–54,000,000 on chromosome 7, we extracted single nucleotide polymorphisms identified through whole exome sequencing that were heterozygous in the patient and homozygous in the mother. Maternal ratio was calculated based on the read counts.
